# A Strange Twist

**DOI:** 10.5811/cpcem.2022.4.56344

**Published:** 2022-07-27

**Authors:** Annete O’Connell, Rodrigo Kong, Rohan Biswas, Josh Greenstein, Barry Hahn

**Affiliations:** * Staten Island University Hospital, Department of Emergency Medicine, Staten Island, NY; † Staten Island University Hospital, Department of Radiology, Staten Island, NY

**Keywords:** fallopian tube, pelvic pain, torsion

## Abstract

**Case Presentation:**

A 16-year-old female presented to the emergency department with acute onset of right lower quadrant abdominal pain for several hours. The patient was afebrile and physical examination was notable for isolated tenderness in the right lower quadrant. Ultrasound and computed tomography demonstrated an adnexal cystic structure. Pelvic magnetic resonance imaging was ordered to better characterize the pathology.

**Discussion:**

Isolated fallopian tube torsion is an uncommon entity requiring prompt surgical intervention. Recognition and appropriate management are essential.

## CASE PRESENTATION

**Image 1:**
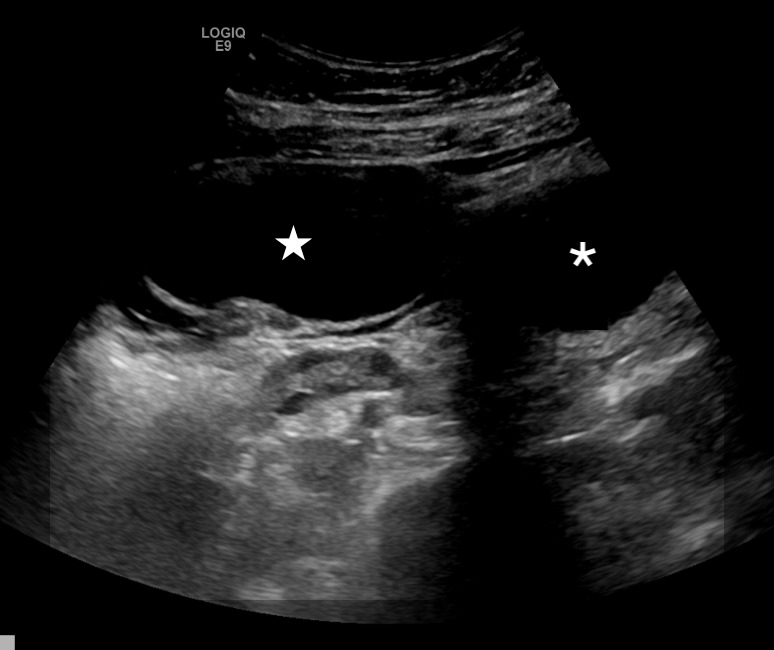
Transabdominal ultrasound image obtained with a curvilinear 5-megahertz transducer demonstrates a 7-centimeter cystic mass (star) in the right adnexa near midline, adjacent to the bladder (asterisk).

A 16-year-old nulliparous, sexually active female with a history of type 2 diabetes mellitus presented to the emergency department with acute onset of non-radiating right lower quadrant pain and tenderness to palpation for several hours. She reported nausea but denied fever, vomiting, diarrhea, anorexia, urinary symptoms or vaginal discharge. Vital signs were blood pressure 135/77 milliliters mercury (mmHg), heart rate 103 beats per minute, and temperature 37.2°C. Physical examination was significant for tenderness to palpation in the right lower abdomen without peritoneal signs. A pelvic examination was unremarkable. Urine pregnancy was negative. Pelvic ultrasound demonstrated an 8 × 8 × 5 centimeter (cm) right adnexal cystic structure, normal-sized ovaries without edema, and normal Doppler flow (Image 1).

**Image 2:**
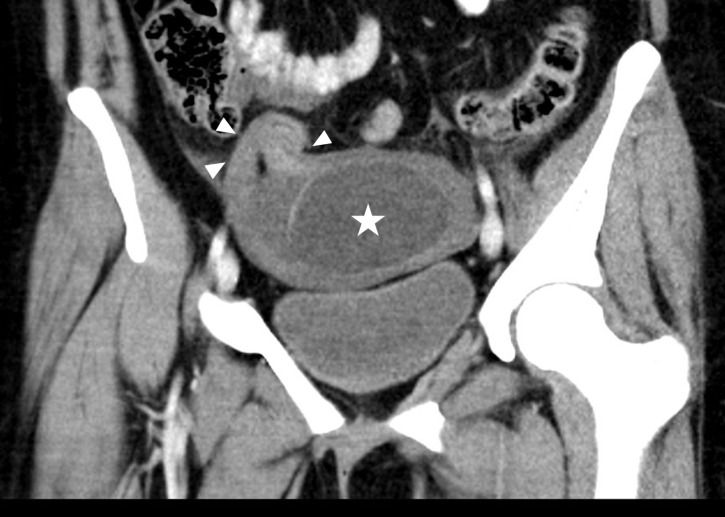
Coronal computed tomography of the pelvis shows the 7-centimeter cystic structure (star) within the pelvis with surrounding fluid. Along the right side of the cystic structure there is extension into the location of the area of the right fallopian tube (arrowheads).

This finding prompted computed tomography (CT) with intravenous contrast to delineate the pathology further. Computed tomography revealed a large cystic structure within the pelvis, distinct from the right ovary (Image 2).

**Image 3:**
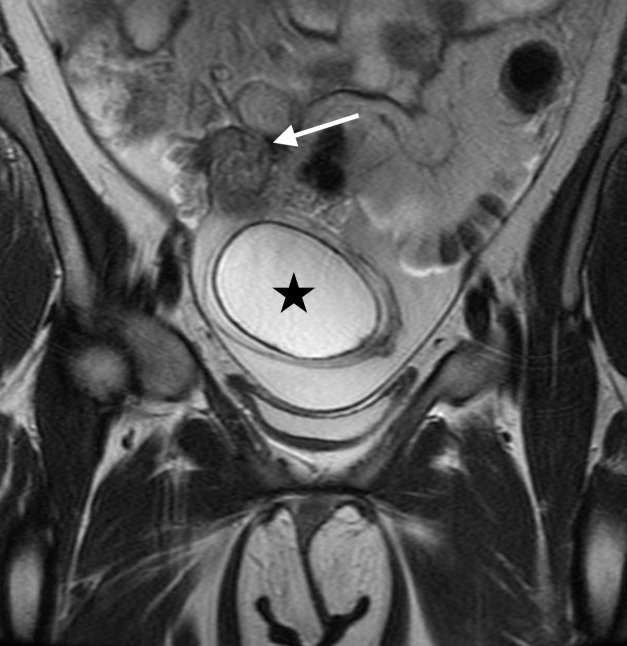
Coronal T2 weighted magnetic resonance imaging of the pelvis demonstrates a 7-centimeter fluid signal tubal mass (star) with a twisted appearance of the torsed right fallopian tube (arrow).

Emergent pelvic magnetic resonance imaging (MRI) with and without intravenous contrast was subsequently ordered to better characterize the masses (Image 3).

## DISCUSSION

Isolated fallopian tube torsion (IFTT) is the rotation of the fallopian tube on itself or around its ligamentous supporting structures. This process is uncommon and typically co-occurs with torsion of the ovary, which is termed adnexal or tubo-ovarian torsion. The reported incidence of IFTT ranges from 1:500,000 to 1:1,500,000, without a defined predilection to a specific age group.^1^ Proposed risk factors for IFTT include pathology of the fallopian tube, endometriosis, and adhesions.^1^,^2^

Patients typically report sudden onset of sharp lower abdominal pain, nausea and vomiting, and localized tenderness.^3^ Differential diagnoses include ovarian torsion, adnexal torsion, ectopic pregnancy, ruptured cyst, tubo-ovarian abscess, and appendicitis. Patients have ipsilateral hydrosalpinx or para-ovarian cysts.^1^,^3^–^4^ Para-ovarian, or paratubal cysts, are encapsulated, fluid-filled sacs that form near an ovary or fallopian tube but do not adhere to any internal organ.

**CPC-EM Capsule**

What do we already know about this clinical entity?

*Isolated fallopian tube torsion is an uncommon entity requiring prompt surgical intervention*.

What is the major impact of the image(s)?

*Diagnosis requires a high index of suspicion supported by advanced imaging*.

How might this improve emergency medicine practice?

*Recognition and appropriate management are essential*.

Pelvic ultrasound is the initial imaging method for evaluating most gynecological pathology. Sonographic findings of IFTT include fallopian tube dilation with wall thickening in the setting of normal-appearing ovaries.^2^ Computed tomography and MRI may aid in the diagnosis. On CT, a mass between the uterus and the ovary is a sensitive (97%) and specific (81%) feature in women with adnexal torsion. Still, no consistent characteristic has been described for IFTT.^2^ Magnetic resonance imaging is preferable for sparing radiation exposure but may not always be readily available in all EDs. Definitive treatment is surgical detorsion. Salpingectomy is controversial and may hinder future fertility.^1^–^4^

Intraoperatively, the patient was found to have right hydrosalpinx and bilateral paratubal cysts, right-sided measuring 5 cm in maximum dimension, and left-sided measuring 7 × 5 × 1 cm. The patient underwent right salpingectomy, fallopian tube detorsion, and bilateral cystectomy without complications. The pathology report was negative for malignancy, and tumor markers were within normal limits. The patient remained asymptomatic at two-week follow-up.

## References

[1] Casey RK, Damle LF, Gomez-Lobo V (2013). Isolated fallopian tube torsion in pediatric and adolescent females: a retrospective review of 15 cases at a single institution. J Pediatr Adolesc Gynecol.

[2] Delacroix C, Hcini N, Vintejoux E (2021). Isolated tubal twist: a case series of a rare event occurring at different times in reproductive life. Int J Surg Case Rep.

[3] Hagege R, Sharvit M, Hamou B (2021). Isolated fallopian tube torsion: an under-diagnosed entity with debatable management. J Minim Invasive Gynecol.

[4] Bertozzi M, Noviello C, Molinaro F (2020). Isolated fallopian tube torsion in pediatric age: an italian multicenter retrospective study. J Pediatr Surg.

